# Sedentary behaviour as a risk factor for cardiovascular diseases in pediatric age

**DOI:** 10.1186/1824-7288-40-S1-A22

**Published:** 2014-08-11

**Authors:** Chiara Mameli, Valentina Fabiano, Gian Vincenzo Zuccotti

**Affiliations:** 1 Department of Pediatrics, L.Sacco Hospital, University of Milan, Italy

## 

The term “sedentary” has historically been used to refer to individuals with low physical activity levels or not meeting some criterion level of physical activity. According to the recent findings, sedentary behaviour has been defined not simply as the absence of moderate-to-vigorous physical activity, but as a class of behaviours (e.g. sitting, watching TV or playing video games) characterized by little physical movement and low energy expenditure (≤ 1.5 metabolic equivalents –METs-) [1].

Recent evidences suggest that sedentary is a distinct risk factors for chronic diseases including cardiometabolic diseases [2].

In Western countries sedentary time increased dramatically since 1960s [3]. Schools, homes, and public spaces have been re-engineered minimizing physical activity and contributing to reduce children independent mobility. [4] Moreover the new media (eg. computer and videogames) occupy a high proportion of leisure-time of children and youth.

Sedentary behavior can be assessed using both indirect (questionnaires) and direct measurement tools (accelerometers). These methods assess different aspects of sedentary: while self- and proxy-report questionnaires mainly focused on common sedentary behaviours (eg. watching TV), accelerometers better estimate the individual’s total sedentary profile. Therefore in clinical studies the concurrent use of both strategies should be encouraged [5].

In pediatric age, studies have demonstrated that spending excessive time engaging in sedentary behaviors, independent from overall physical activity levels, is adversely associated with adiposity and other cardiometabolic risk factors such as lipid profile, blood pressure and central obesity [5,6]. In particular, the self-reported screen time appears to be more strongly associated with these risk factors than the total sedentary time assessed by accelerometers. One potential explanation could be that certain forms of sedentary activities (TV viewing and more in general screen-based activities) promote the excess of food intake and thus a positive energy balance. Research community should continue to accrue knowledge about the impact of sedentary on health in the pediatric age. Efforts should be done to promote deliberate physical activity and to decrease sedentary behaviours in order to maximize health benefits since childhood.
